# Preoperative circulating tumor cells level is associated with lymph node metastasis in patients with unifocal papillary thyroid carcinoma

**DOI:** 10.1186/s12957-025-03702-8

**Published:** 2025-02-11

**Authors:** Yihua Gu, Ming Yu, Jiaqin Deng, Yeqian Lai

**Affiliations:** Department of Thyroid Surgery, Meizhou People’s Hospital, Meizhou Academy of Medical Sciences, Meizhou, China

**Keywords:** Circulating tumor cell, Papillary thyroid carcinoma, Unifocal, Lymph node metastasis

## Abstract

**Objective:**

Unifocal papillary thyroid carcinoma (PTC) refers to thyroid cancer that has only one isolated lesion, it has also the possibility of lymph node metastasis (LNM). Circulating tumor cell (CTC) has been used to assist in the assessment of tumor progression, but the relationship between CTCs levels and LNM in unifocal PTC patients is unclear.

**Methods:**

The clinical records (age, gender, Hashimoto’s thyroiditis, thyroid function, tumor size, invaded capsule (thyroid cancer penetrating the capsule), clinical stage, and LNM) of unifocal PTC patients in Meizhou People’s Hospital were analyzed retrospectively. Receiver operating characteristic (ROC) curve analysis was used to determine the cutoff value of CTCs levels to distinguish LNM. The relationship between CTCs level and clinical features was analyzed. Logistic regression analysis was used to evaluate the relationship between CTCs and LNM.

**Results:**

A total of 507 unifocal PTC patients were included, and 198(39.1%) patients with LNM. The critical value of CTCs was 9.25 FU/3mL by ROC analysis, and 288(56.8%) unifocal PTC patients with preoperative CTC-positive(≥ 9.25 FU/3mL). The patients with positive CTCs had higher proportions of normal thyroid function (91.3% vs. 84.5%, *p* = 0.018), and LNM (44.1% vs. 32.4%, *p =* 0.008) than patients with negative. High preoperative CTCs level (≥ 9.25/<9.25 FU/3mL, odds ratio(OR): 1.653, 95% confidence interval(CI): 1.115–2.451, *p* = 0.012), tumor size > 1 cm (OR: 3.189, 95% CI: 2.069–4.913, *p* < 0.001), and invaded capsule (OR: 1.521, 95% CI: 1.005–2.302, *p* = 0.047) were associated with LNM among unifocal PTC in multivariate logistic regression analysis.

**Conclusions:**

High preoperative CTCs level (≥ 9.25 FU/3mL), tumor size > 1 cm, and invaded capsule were associated with LNM among unifocal PTC.

## Introduction

Malignant tumor is the main cause of death of Chinese residents [1], and most of the death of malignant tumor is caused by tumor metastasis [2, 3]. The process of tumor metastasis is roughly as follows: tumor cells break through the vascular barrier to enter the blood circulation, survive in the blood circulation system, break through the vascular barrier to enter specific tissue sites, proliferate and eventually form metastases [4, 5]. Tumor cells that survive in circulation after breaking through the blood vessel barrier are called circulating tumor cells (CTCs). CTCs as a tumor biomarker, are tumor cells that are released into the circulation of peripheral blood from the primary site or metastasis site spontaneously or due to clinical procedures [6]. At present, CTCs have been used in early diagnosis and screening of tumors, evaluating stages, monitoring postoperative changes, metastasis and recurrence, verifying treatment effects, and determining drug sensitivity [6–8].

Thyroid cancer is one of the most common endocrine cancers of the head and neck [9, 10], and the latest statistics show that there are about 600,000 new cases of thyroid cancer worldwide every year, with a higher incidence in women than in men [11]. According to the origin and differentiation of the tumor, thyroid cancer can be divided into four categories: papillary thyroid cancer (PTC), follicular thyroid cancer (FTC), medullary thyroid cancer (MTC), and undifferentiated thyroid cancer (UTC) [12, 13]. PTC accounts for about 85-90% of all thyroid tumors, and its prognosis is good, with an early 10-year survival rate of up to 90% [14]. PTC is well differentiated and has low malignancy, and is often considered as an inert tumor; however, it is prone to lymph node metastasis [15–18]. Lymph node metastasis (LNM) occurs in about 10-15% of PTC patients during the development of the disease, and the emergence of metastatic lesions will greatly reduce the quality of life of PTC patients and affect the prognosis, and the mortality rate of these PTC patients in the late 10 years will increase significantly to 70% [19]. As the gold standard for the diagnosis of PTC, ultrasound (US) guided fine needle aspiration cytology (UG-FNAC) is limited by its low sensitivity and invasive methods [20, 21].

There is evidence that LNM in patients with PTC is adverse to prognosis, especially in patients with Hashimoto’s thyroiditis, large tumor diameter, and envelope invasion [22–24]. Unifocal PTC refers to thyroid cancer that has only one isolated lesion on its anatomy, and multifocal PTC is PTC with two or more anatomically separated lesions in the thyroid gland [25, 26]. There are differences in the risk of progression and the likelihood of poor prognosis between multifocal and unifocal tumors [27, 28]. Whether the clinician performs prophylactic lymph node dissection during surgery and the extent of the dissection depends on the assessment of the patient’s risk of LNM [29–31]. It is necessary to investigate the related factors of LNM in unifocal PTC to provide evidence for the scope of intraoperative cervical lymph node dissection. Several studies have found that CTCs are associated with the prognosis [32, 33] and distant metastasis [32] of thyroid cancer. However, it is unclear whether CTCs are associated with LNM in patients with unifocal PTC. The purpose of this study is to study the relationship between them. It is expected to provide valuable reference data for the role of CTCs in the risk assessment of LNM in unifocal PTC.

## Materials and methods

### Subjects

**Fig. 1:**
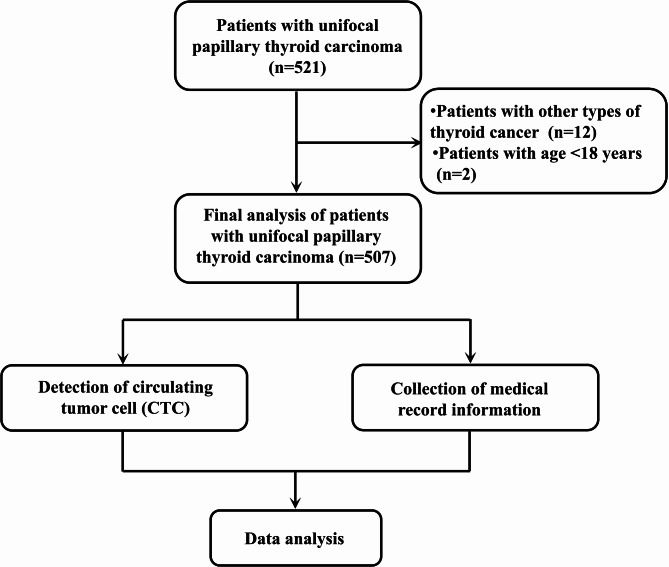
The flow chart of the present study

This study retrospectively analyzed the clinical records of 507 patients with unifocal PTC who were hospitalized in Meizhou People’s Hospital from June 2021 to April 2023. Inclusion criteria of patients were as follows: (1) age ≥ 18 years; (2) the initial thyroid surgery was performed and the postoperative pathologic findings proved to be unifocal PTC; (3) patients without other tumors; and (4) the patients’ medical records were complete. The exclusion criteria were as follows: (1) age < 18 years; (2) patients with previous history of neck surgery or exposure; (3) patients with other types of thyroid cancer; (4) patients with other malignant tumors; and (5) patients with multifocal PTC. This study was supported by the Ethics Committee of the Meizhou People’s Hospital. The flowchart of this study is shown in Fig. 1.

### Data collection

Clinical medical records of the unifocal PTC patients were collected, such as age, gender, preoperative CTCs, Hashimoto’s thyroiditis, thyroid function, maximum lesion diameter, invaded capsule, clinical stage, and LNM. The tumor size group was divided into two groups: PTC with maximum lesion diameter ≤ 1 cm and maximum lesion diameter > 1 cm [34, 35].

Three milliliter (ml) peripheral venous blood was collected from each tester into 6 ml ethylenediaminetetraacetic acid (EDTA)-containing test tubes for folate receptor-positive circulating tumor cells (FR + CTCs) analysis. Peripheral blood CTCs were detected by reverse transcription-polymerase chain reaction (RT-PCR) technique using the CytoploRare Kit (Genosaber Biotech, Shanghai, China). Specifically, the red blood cells and the vast majority of white blood cells were depleted using the negative enrichment method to obtain folate receptor-positive cells. The folate receptor-positive cells were then labeled with specific small molecule probes. Finally, the oligonucleotides in folic acid receptor binding small molecule probes were quantitatively detected by polymerase chain reaction (PCR) using a specific primer designed for small molecule probes and Taqman fluorescent probes. Folate receptor Unit (FU) per 3mL (FU/3mL) as defined in the manufacturer’s manual, was used to represent the level of FR + CTC in 3 mL of peripheral blood. In this study, receiver operating characteristic (ROC) curve analysis was used to determine the optimal cutoff value of CTCs levels to distinguish LNM. According to the ROC analysis, CTCs ≥ cutoff value is considered to be positive for CTCs levels, and CTCs < cutoff value is negative.

### Statistical analysis

SPSS statistical software (version 26.0, IBM Inc., USA) was used for data analysis. Chi-square test or Fisher’s exact test were used to evaluate the relationship between different CTCs levels and clinical features of unifocal PTC patients. Univariate and multivariate logistic regression analyses were used to evaluate the relationship between CTCs and LNM in patients with unifocal PTC, based on estimating the odds ratios (OR) and their 95% confidence intervals (CIs). And age, gender, Hashimoto’s thyroiditis, thyroid function, maximum lesion diameter, invaded capsule, and clinical stage were selected as covariates in the multivariate logistic regression analysis for the association between CTCs and LNM. *p* < 0.05 was set as statistically significant.

## Results

### Clinicopathological features of patients with unifocal PTC

There were 393 cases (77.5%) were < 55 years old and 114 cases (22.5%) were ≥ 55 years old; and 100 male patients (19.7%) and 407 female patients (80.3%) among the 507 patients with unifocal PTC. There were 125 (24.7%), 59 (11.6%), 151 (29.8%), and 190 (37.5%) patients with Hashimoto’s thyroiditis, abnormal thyroid function, maximum lesion diameter > 1 cm, and invaded capsule, respectively. There were 198 (39.1%) patients with LNM. When LNM was taken as the endpoint of CTCs, the critical value was 9.25 FU/3mL (sensitivity = 64.1%, specificity = 47.9%, area under the ROC curve (AUC) = 0.531). There were 288 (56.8%) unifocal PTC patients with preoperative positive CTCs (CTCs ≥ 9.25 FU/3mL), and 219 (43.2%) with negative CTCs (CTCs ≥ 9.25 FU/3mL) (Table 1).

**Table 1:** The clinicopathological features of patients with unifocal PTC

Clinicopathological features	PTC patients (*n* = 507)
Age (Years)	
< 55, n (%)	393 (77.5%)
≥ 55, n (%)	114 (22.5%)
Gender	
Male, n (%)	100 (19.7%)
Female, n (%)	407 (80.3%)
Hashimoto’s thyroiditis	
No, n (%)	382 (75.3%)
Yes, n (%)	125 (24.7%)
Thyroid function	
Normal, n (%)	448 (88.4%)
Abnormal, n (%)	59 (11.6%)
Maximum lesion diameter	
≤ 1 cm, n (%)	356 (70.2%)
> 1 cm, n (%)	151 (29.8%)
Invaded capsule	
No, n (%)	317 (62.5%)
Yes, n (%)	190 (37.5%)
T stage	
T1-T2, n (%)	469 (92.5%)
T3-T4, n (%)	38 (7.5%)
Lymph node metastasis	
No, n (%)	309 (60.9%)
Yes, n (%)	198 (39.1%)
Preoperative circulating tumor cells (CTCs) (FU/3mL)	
Negative (< 9.25)	219 (43.2%)
Positive (≥ 9.25)	288 (56.8%)

PTC, papillary thyroid carcinoma; CTC, circulating tumor cell; FU, folate receptor unit

### Comparison of clinicopathological features among unifocal PTC patients with negative or positive CTCs

The unifocal PTC patients with positive CTCs had higher proportions of normal thyroid function (91.3% vs. 84.5%, *p* = 0.018), and LNM (44.1% vs. 32.4%, *p =* 0.008) than patients with negative CTCs. There was no statistically significant difference in distributions of age and gender, and proportions of Hashimoto’s thyroiditis, maximum lesion diameter > 1 cm, invaded capsule, and T stage between patients with negative and positive CTCs (Table 2).

**Table 2:** Comparison of clinicopathological features among unifocal PTC patients with negative or positive CTCs

Clinicopathological features	CTCs (FU/3mL)		*p* values
	< 9.25 (*n* = 219)	≥ 9.25 (*n* = 288)	
Age (Years)			
< 55, n (%)	164(74.9%)	229(79.5%)	0.238
≥ 55, n (%)	55(25.1%)	59(20.5%)	
Gender			
Male, n (%)	37(16.9%)	63(21.9%)	0.177
Female, n (%)	182(83.1%)	225(78.1%)	
Hashimoto’s thyroiditis			
No, n (%)	167(76.3%)	215(74.7%)	0.755
Yes, n (%)	52(23.7%)	73(25.3%)	
Thyroid function			
Normal, n (%)	185(84.5%)	263(91.3%)	0.018
Abnormal, n (%)	34(15.5%)	25(8.7%)	
Maximum lesion diameter			
≤ 1 cm, n (%)	156(71.2%)	200(69.4%)	0.696
> 1 cm, n (%)	63(28.8%)	88(30.6%)	
Invaded capsule			
No, n (%)	135(61.6%)	182(63.2%)	0.781
Yes, n (%)	84(38.4%)	106(36.8%)	
T stage			
T1-T2, n (%)	202(92.2%)	267(92.7%)	0.866
T3-T4, n (%)	17(7.8%)	21(7.3%)	
Lymph node metastasis			
No, n (%)	148(67.6%)	161(55.9%)	0.008
Yes, n (%)	71(32.4%)	127(44.1%)	

PTC, papillary thyroid carcinoma; CTC, circulating tumor cell; FU, folate receptor unit

### Comparison of clinical features of different CTCs levels among unifocal PTC patients with and without LNM, respectively

In unifocal PTC patients without LNM, there was no statistically significant difference in distributions of age and gender, and proportions of Hashimoto’s thyroiditis, abnormal thyroid function, maximum lesion diameter > 1 cm, invaded capsule, and T stage between patients with negative and positive CTCs. In unifocal PTC patients with LNM, patients with positive CTCs had a lower proportion of maximum lesion diameter > 1 cm than that in patients with negative CTCs (40.9% vs. 59.2%, *p* = 0.018). There were no significant difference in other features between different CTC levels in patients with or without lymph node metastasis (Table 3).

**Table 3:** Comparison of clinicopathological features of different CTCs levels among unifocal PTC patients with and without lymph node metastasis, respectively

	Lymph node metastasis					
Clinicopathological features	No (*n* = 309)			Yes (*n* = 198)		
	CTCs < 9.25 (*n* = 148)	CTCs ≥ 9.25 (*n* = 161)	*p* values	CTCs < 9.25 (*n* = 71)	CTCs ≥ 9.25 (*n* = 127)	*p* values
Age (Years)						
< 55, n (%)	110(74.3%)	125(77.6%)	0.508	54(76.1%)	104(81.9%)	0.359
≥ 55, n (%)	38(25.7%)	36(22.4%)		17(23.9%)	23(18.1%)	
Gender						
Male, n (%)	24(16.2%)	31(19.3%)	0.552	13(18.3%)	32(25.2%)	0.294
Female, n (%)	124(83.8%)	130(80.7%)		58(81.7%)	95(74.8%)	
Hashimoto’s thyroiditis						
No, n (%)	116(78.4%)	116(72.0%)	0.236	51(71.8%)	99(78.0%)	0.388
Yes, n (%)	32(21.6%)	45(28.0%)		20(28.2%)	28(22.0%)	
Thyroid function						
Normal, n (%)	122(82.4%)	145(90.1%)	0.067	63(88.7%)	118(92.9%)	0.428
Abnormal, n (%)	26(17.6%)	16(9.9%)		8(11.3%)	9(7.1%)	
Maximum lesion diameter						
≤ 1 cm, n (%)	127(85.8%)	125(77.6%)	0.078	29(40.8%)	75(59.1%)	0.018
> 1 cm, n (%)	21(14.2%)	36(22.4%)		42(59.2%)	52(40.9%)	
Invaded capsule						
No, n (%)	98(66.2%)	117(72.7%)	0.265	37(52.1%)	65(51.2%)	1.000
Yes, n (%)	50(33.8%)	44(27.3%)		34(47.9%)	62(48.8%)	
T stage						
T1-T2, n (%)	144(97.3%)	154(95.7%)	0.546	58(81.7%)	113(89.0%)	0.195
T3-T4, n (%)	4(2.7%)	7(4.3%)		13(18.3%)	14(11.0%)	

PTC, papillary thyroid carcinoma; CTC, circulating tumor cell

### Logistic regression analysis of risk factors of LNM in unifocal PTC

High preoperative CTCs level (≥ 9.25 vs. <9.25 FU/3mL, odds ratio (OR): 1.644, 95% confidence interval (CI): 1.140–2.372, *p* = 0.008), maximum lesion diameter > 1 cm (> 1 cm vs. ≤1 cm, OR: 3.996, 95% CI: 2.677–5.965, *p* < 0.001), T3-T4 stage (T3-T4 vs. T1-T2, OR: 4.278, 95% CI: 2.070–8.839, *p* < 0.001), and invaded capsule (OR: 2.153, 95% CI: 1.488–3.114, *p* < 0.001) were associated with LNM among unifocal PTC in univariate logistic regression analysis. In addition, high preoperative CTCs level (≥ 9.25 vs. <9.25 FU/3mL, OR: 1.653, 95% CI: 1.115–2.451, *p* = 0.012), maximum lesion diameter > 1 cm (> 1 cm vs. ≤1 cm, OR: 3.189, 95% CI: 2.069–4.913, *p* < 0.001), and invaded capsule (OR: 1.521, 95% CI: 1.005–2.302, *p* = 0.047) were associated with LNM among unifocal PTC in multivariate logistic regression analysis (Table 4).

**Table 4:** Logistic regression analysis of risk factors of lymph node metastasis in unifocal PTC

Variables	Univariate		Multivariate	
	OR (95% CI)	*p* values	OR (95% CI)	*p* values
Preoperative CTCs (≥ 9.25 vs. <9.25, FU/3mL)	1.644 (1.140–2.372)	0.008	1.653 (1.115–2.451)	0.012
Age (< 55 vs. ≥55, years old)	1.244 (0.806–1.920)	0.325	1.390 (0.864–2.235)	0.174
Gender (male vs. female)	1.358 (0.873–2.113)	0.175	1.304 (0.808–2.106)	0.277
Hashimoto’s thyroiditis (yes vs. no)	0.964 (0.637–1.460)	0.863	0.966 (0.614–1.517)	0.879
Thyroid function (abnormal vs. normal)	0.597 (0.330–1.082)	0.089	0.718 (0.379–1.357)	0.307
Maximum lesion diameter (> 1 cm vs. ≤1 cm)	3.996 (2.677–5.965)	< 0.001	3.189 (2.069–4.913)	< 0.001
T stage (T3-T4 vs. T1-T2)	4.278 (2.070–8.839)	< 0.001	1.974 (0.874–4.461)	0.102
Invaded capsule (yes vs. no)	2.153 (1.488–3.114)	< 0.001	1.521 (1.005–2.302)	0.047

PTC, papillary thyroid carcinoma; CTC, circulating tumor cell; FU, folate receptor unit; OR, odds ratio; CI, confidence interval

## Discussion

There is evidence that LNM in patients with thyroid cancer can be adverse to survival, especially in patients with large focal diameter and extragadenial invasion. There are more aggressive cell histological features in multifocal tumors than in unifocal ones [36]. The risk of LNM is higher in patients with multifocal thyroid cancer than in patients with unifocal thyroid cancer [37]. Therefore, the purpose of this study was to explore the risk factors for LNM of unifocal PTC through clinical and pathological characteristics, and to provide data reference for clinical standardized treatment. In this study, high preoperative CTCs level (≥ 9.25 FU/3mL), maximum lesion diameter > 1 cm, and invaded capsule were associated with LNM among unifocal PTC.

In recent years, liquid biopsy technology for detecting circulating tumor cells has attracted much attention [38, 39]. Some studies have found that CTC is an effective marker for the diagnosis [40], prognosis [33, 41], and recurrence and distant metastasis [42] of thyroid cancer. Yu et al. found that high CTCs levels are associated with LNM in papillary thyroid microcarcinoma (PTMC) [43]. The relationship between CTC and LNM in patients with unifocal PTC remains unclear. The results of this study also suggested that high preoperative CTCs level (≥ 9.25/FU/3mL) was associated with LNM in unifocal PTC. In terms of mechanism, CTCs in thyroid patients are mainly cells with mesenchymal phenotype [44, 45]. CTCs undergo mesenchymal transformation [46–48] and through interactions with blood cells and immune cells [49, 50] to invade the lymphatic and circulatory system. The presence of high CTCs levels means that more CTCs are likely to invade the lymphatic system. The prevalence of LNM in PTC patients varied among different studies, ranging from 13.94–63.72% [51]. LNM affects the prognosis of patients, and it is of great significance to explore the risk factors for predicting LNM in PTC patients. With the establishment and application of commercial CTCs detection systems and the gradual reduction of detection costs, CTC has become a predictor of LNM with clinical application potential. However, in this study, the relatively low AUC value (0.531) suggests limited predictive accuracy for CTCs alone. Therefore, CTC-based risk prediction of LNM in unifocal PTC patients needs to be combined with other indicators (such as gender, age, tumor size, capsule invasion, and so on) for comprehensive assessment.

Larger lesion diameter was a high risk factor for LNM in unifocal PTC patients [52, 53]. Hei et al. found that tumor size, and invaded capsule were associated with lateral neck metastasis in patients with unifocal PTC [54]. Huang et al. suggested that tumor size > 7 mm was a risk factor for lateral lymph node metastasis (LLNM) in cN0 unifocal PTMC [53]. In both men and women, tumor size was a factor affecting central lymph node metastasis (CLNM) in cN0 unifocal PTC patients in another study [55]. In addition, some studies suggest that LNM of thyroid cancer is related to invaded capsule [56–58]. Invaded capsule often indicates malignant proliferation and local invasion of tumors, and is an important risk factor for LNM [59, 60]. The confidence interval for invaded capsule (95% CI: 1.005–2.299) is relatively wide, and it may occur because the data points are scattered relative to the regression line. It may be due to the presence of outliers or other factors that affect the stability of the model. The association between invaded capsule and the risk of LNM in unifocal PTC needs more studies to support.

Regarding the relationship between age and thyroid cancer LNM, some studies have shown that older patients have a higher risk of developing LNM [61], while some studies have suggested that younger patients have a higher risk of developing LNM [51, 62]. Yu et al. found that younger age (< 55 years) was a high risk factor for LNM in unifocal PTC patients [52]. The relationship between age and LNM in unifocal PTC patients was not found in this study. PTC is more common in women than men [63]. Several studies have shown that the incidence of PTC in women is significantly related to estrogen levels [64, 65], while male patients are more susceptible to unhealthy lifestyle and harmful environment [66, 67]. Moreover, some studies suggest that men with PTC are more likely to develop LNM [68–71]. In unifocal PTC patients, some studies have identified that male was a high risk factor for LNM [52, 72]. The relationship between gender and LNM was not found in this study. Some studies suggest that Hashimoto’s thyroiditis is closely related to the occurrence of thyroid cancer. Several studies suggest that Hashimoto’s thyroiditis is related to LNM of PTC [73, 74]. It is considered that Hashimoto’s thyroiditis, as an autoimmune inflammatory disease, can mediate autoimmune damage through both inflammatory and immune mechanisms, making it a precancerous lesion [75]. However, other studies have suggested that Hashimoto’s thyroiditis reduces the risk of LNM of PTC [76–78]. The relationship between Hashimoto’s thyroiditis and LNM in unifocal PTC patients was not found in this study.

At present, the differences between surgical choice, prognosis, and risk of recurrence in patients with unifocal and multifocal PTC have not been consistently understood [79–82]. In this study, high preoperative CTCs level (≥ 9.25/<9.25 FU/3mL), maximum lesion diameter > 1 cm, and invaded capsule were associated with LNM among unifocal PTC. The findings suggest that comprehensive consideration of these indicators can predict whether there is LNM in unifocal PTC patients, and it provide reference data for the selection of lymph node dissection scope in clinical surgery. This study provides valuable reference data for the evaluation of LNM of unifocal PTC. But it has some shortcomings: (1) this study is a single-center retrospective study with limited sample size and lack of external data validation, so multi-center, large-sample studies are still needed to confirm this result; (2) a predictive model for central LNM of unifocal PTC based on multimodal ultrasound features has been reported [83], the ultrasound characteristics of the patients were not included in this study; and (3) this study lacks the classification analysis of PTC subtypes, and it may be more clinical significance to classify PTC of different subtypes.

## Conclusions

High preoperative CTCs level (≥ 9.25 FU/3mL), maximum lesion diameter > 1 cm, and invaded capsule were associated with LNM among unifocal PTC. In other words, unifocal PTC patients with maximum lesion diameter > 1 cm and had invaded capsule might be prone to LNM when preoperative CTCs level ≥ 9.25 FU/3mL. It provides valuable reference data for the risk assessment of LNM in unifocal PTC. The liquid biopsy technology of CTC detection has the advantages of good patient compliance, easy sample acquisition, and effective real-time monitoring, which has potential application value.

## Data Availability

No datasets were generated or analysed during the current study.
